# Interictal Cognitive Performance in Children and Adolescents With Primary Headache: A Narrative Review

**DOI:** 10.3389/fneur.2022.898626

**Published:** 2022-07-13

**Authors:** Samuela Tarantino, Martina Proietti Checchi, Laura Papetti, Fabiana Ursitti, Giorgia Sforza, Michela Ada Noris Ferilli, Romina Moavero, Gabriele Monte, Teresa Grimaldi Capitello, Federico Vigevano, Massimiliano Valeriani

**Affiliations:** ^1^Unit of Clinical Psychology, Bambino Gesù Children's Hospital, Istituto di Ricovero e Cura a Carattere Scientifico (IRCCS), Rome, Italy; ^2^Department of Neuroscience, Headache Center, Bambino Gesù Children's Hospital, Istituto di Ricovero e Cura a Carattere Scientifico (IRCCS), Rome, Italy; ^3^Child Neurology and Psychiatry Unit, Tor Vergata University of Rome, Rome, Italy; ^4^Center for Sensory-Motor Interaction, Aalborg University, Aalborg, Denmark

**Keywords:** headache, children, neuropsychology, cognitive performance, intelligence, memory, attention

## Abstract

Primary headache is a very common and disabling disease. The burden of pain and recurrent attacks may lead to a poor quality of life, anxiety and depression. An increased risk of low functioning and curricular performances in young patients with primary headache has been described. The mechanisms underlying the relationship between migraine and poor school achievement may be various and could be a reflection of weak cognitive skills. Data concerning the cognitive functioning in the free pain interval in pediatric age are under-investigated and results are far from conclusive. The present review article suggests that, though considered a benign disease, pediatric migraine may be associated to altered neuropsychological functioning in the interictal phase. Although children and adolescents with migraine generally have a normal intelligence, they may show a not homogeneous cognitive profile, characterized by possible difficulties in verbal skills, in particular comprehension abilities. Pediatric primary headache may present altered neuropsychological functioning involving attentional resources, processing speed and memory, particularly verbal memory. Given the impact that this disease can have on school performance and the tendency to persist from childhood to adulthood, a cognitive screening in young patients affected by primary headache is pivotal. Additional neuropsychological research using more homogenous methods is needed.

## Introduction

Primary headache is a very common and disabling disease (1, 2). Migraine is characterized by moderate to severe pulsating pain attacks lasting even 72 h and aggravated by routine physical activity (3). In pediatric age, pain is more often bilateral than in adults. Migraine pain is often associated with nausea and/or vomiting, phonophobia and photophobia. In migraine with aura, pain may be accompanied or preceded by reversible visual, sensory or motor symptoms (3). Bilateral and pressing/tightening attacks of moderate or mild intensity pain are the main features of tension-type headache (TTH) (3). The burden of pain and recurrent attacks may lead to a poor quality of life, social isolation, anxiety and depression. The impact of headache on academic achievement and school attendance has been described since'80. In an early study, Leviton et al. showed that, out of 150 elementary-school children with headache referred to his center, ~40% had academic difficulties (4). Over the time, several studies highlighted an increased risk of low functioning and curricular performances in young patients with migraine, even compared with patients with cardiomyopathy, cancer and diabetes (5–10). Some vulnerability factors, such as the frequency and intensity of pain and the presence of associated symptoms, have been identified as determinants in the association between migraine and academic failure (10). The mechanisms underlying the relationship between migraine and poor school achievement may be various and could also include: (1) absenteeism from school (11); (2) sleep deprivation as a consequence of pain (12); (3) emotional and behavioral symptoms associated with recurrent pain, affecting the learning process (13); (4) reduced attention in the classroom due to pain and unwellness (12).

Poor academic performance in children and adolescents with migraine may also be a reflection of weak cognitive skills (14). Although cognitive difficulties are not considered “core” symptoms of migraine, a low percentage of patients may experience transient and premonitory cognitive symptoms, such as speech or language impairment, as part of typical aura, but also color and face recognition difficulties, and memory abnormalities (3, 15). Transient neuropsychological problems, such as poor ability to concentrate and feeling of distraction, incapacity to think clearly and to deal with multiple tasks, are often described in the ictal phase of the migraine attacks and may contribute to the attack-related disability (16–18). These reversible neuropsychological disturbances have been attributed to the widespread and complex brain modifications and dysfunction underlying the development of the migraine attacks (16). Since transient cognitive symptoms may also persist after the resolution of pain (16, 19), they cannot be merely explained by pain (16). Electrophysiological and functional alterations in cortical functioning during the free pain interval have been reported in both children and adults (20–23), suggesting the presence of interictal cognitive dysfunctions in patients suffering from migraine. Neurophysiological data on adult and pediatric populations, have evidenced altered processing of stimuli in patients with migraine and TTH (17, 18, 24–26). Moreover, several neuro-imaging studies disclosed structural abnormalities of the brain, such as reduced cortical thickness (27–30) and decreased frontal and parietal lobe gray matter density (31), areas of hypoperfusion (32), and focal or generalized atrophies (i.e., temporal lobe) (33), that may represent the anatomical counterpart of the low performance in cognitive tests (34, 35).

Neuropsychological studies in adults found impaired cognitive functions, such as attention, processing speed (reaction time), executive functioning, verbal and visual memory, during the interictal phase of migraine (16, 35, 36). With regard to children and adolescents, data concerning the cognitive functioning in the free pain interval are under-investigated and results are far from conclusive (14). Considering that school functioning is one of the most important domains of children's life and that childhood and adolescence are critical periods for appropriate academic achievements and personal growth, an analysis of cognitive interictal impairment associated with migraine is essential for an appropriate management of children and adolescents with headache.

### Aims

In the present review, we aimed to explore and summarize the current literature on neuropsychological difficulties in children and adolescents with migraine, attempting to describe patients' cognitive profile in the interictal phase of the migraine cycle. Moreover, in order to better understand patients' global needs, we investigated the literature data on the association between headache features, duration of disease and cognitive impairment.

Given the results issued from studies in adult patients with migraine, we hypothesized that: (1) neuropsychological difficulties, particularly those involving memory and attention, may be common also in pediatric migraine, and (2) impairment of cognitive abilities may depend on frequency of migraine attacks and duration of disease.

## Methods

**Figure 1:**
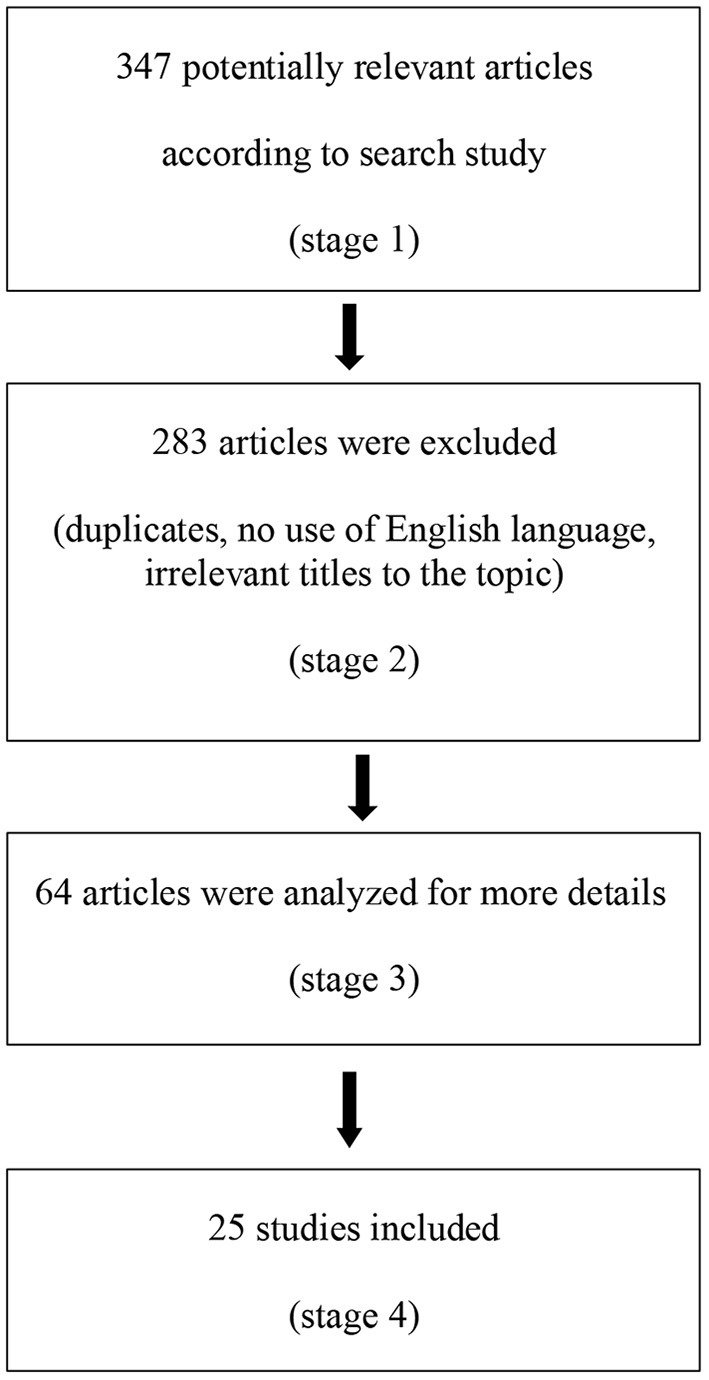
Flow diagram of the study methodology.

Suitable studies were identified using MEDLINE and Web of Science. Our research considered studies published up to December 2021. We considered only papers in the English language. Search terms included “Pediatric migraine” or “Pediatric headache” and “Cognitive performance,” “Cognitive impairment” or “Neuropsychology,” “Intelligence,” “Attention,” “ADHD,” “Memory,” “Language,” “Visuo-spatial,” “Coordination” and “Difficulties” or “Problems.” We considered papers involving subjects of an age ranging from 0 to 18 years. We considered also articles that, though focusing on adults, included subjects <18 years old. Prospective, observational and retrospective studies were considered as well as multicentric studies and clinical trials (Figure 1).

## Results

Using the above described strategy, we identified 25 articles concerning the neuropsychology in pediatric migraine. Among them, 1 was a research letter, 2 were retrospective study, 1a longitudinal research and 2 were population studies. As for multicentric studies, we identified 3 papers. One study evaluated neuropsychological performance in both adults and children. Several different tests were employed to measure neuropsychological abilities across studies.

### General Intelligence and Language

Over the last decades, several authors aimed to study the general intelligence in children with migraine, reporting discrepancies depending on the nature of the tests administered (non-verbal vs. verbal) (Table 1). In 1989, D'Andrea et al. explored the general non-verbal intelligence and reasoning in a small cohort of 20 children with migraine (age range 7–11 years) (37). By using “Raven's Colored Progressive Matrices” (CMP), the authors demonstrated normal cognitive performances and the absence of difference in intelligence levels between patients with migraine and control subjects. Normal general non-verbal intelligence and reasoning were confirmed by Riva et al. (38). In particular, comparing the performance of children suffering from migraine with (n. 17) and without aura (n. 31), the authors did not identify significant differences in the distribution of Progressive Matrices scores between the groups of patients. In a more recent study, Margari et al. evaluated the non-verbal intelligence abilities, including fluid intelligence, attention skills and non-verbal memory, in pediatric patients with primary headache (39). Thirty-five children suffering from migraine (n. 26) or TTH (n. 9) and 23 healthy subjects were evaluated with the cognitive battery “Leiter-3” (57). In line with previous studies, the headache group did not show global cognitive deficits (the IQ mean value in patients was 97.6 ± 11.3). In particular, no statistical significant difference in the non-verbal IQ mean value was found between patients and control group. The study did not find a significant correlation between the attack frequency and duration of headache and Leiter-3 scores.

**Table 1:** Summary of the results issued from the available studies.

**References**	**Sample**	**Neuropsychological domains assessed (Tests used)**	**Main neuropsychological results**
D'Andrea et al. (37)	20 Migraine (7 M; 13 F; age range: 7–11 years) 20 Control subjects (8 M; 12 F; age range: 7–11 years)	- General non-verbal intelligence abilities (CMP) - Memory (Digit Span item of the Wechsler Intelligence Scale; Rey Figures; Logical memory; Ten words learning)	No significant difference in non-verbal intelligence and visual perceptive capacity between patients and control. Short and long-term memories were impaired in migraine group
Riva et al. (38)	31 Migraine without aura (13 M; 18 F, age range 6–16 years) 17 Migraine with aura (6M; 11 F; age range: 9–17 years)	- General non-verbal intelligence (CMP) - Verbal and visuo-spatial memory (Digit Span Test- an item of the Wechsler Intelligence Scale for verbal recall; Corsi Span Test for visuospatial memory); - Divided and selective visual attention (Trail-Making Test and Cancellation test) - Information speed processing (computerized simple visual reaction time task, RT)	Normal non-verbal intelligence, visual, selective and divided attention in both groups of migraine. Dysfunction in the information processing speed (delay in the reaction time) in migraine with and without aura. An influence of the frequency of attacks, but not of the duration of the disease, on the simple reaction time
Margari et al. (39)	24 Migraine without aura 2 Migraine with aura 9 TTH Patients were merged in a group (20 M; 15 F; mean age: 14.89 ± 3.2) 23 Control subjects (14 M; 9 F; mean age: 14.75 ± 3.17)	- General non-verbal intelligence abilities (Leiter International Performance Scale- Third Edition, Leiter-3) - Attention/Memory (Leiter International Performance Scale- Third Edition, Leiter-3)	Normal cognitive performance in patients and control. A significant association between the frequency of attacks and the headache disability with the non-verbal memory and sustained attention
Waldie et al. (34)	114 Migraine 109 TTH Patients were evaluated from 3 to 26 years old, with a mean step of 2 years	- General intelligence abilities (WISC-R) - Receptive and expressive language (The Peabody Picture Vocabulary for receptive language; Illinois Test of Psycholinguistic Ability- Comprehension and Verbal Expression subscales) - Reading (the Burt Word Reading Test) - Academic achievement was estimated from the total scores national exams and a standardized grading system during the final 2 years of high school (ages 15–17)	Subtly, but significantly, lower verbal performance (especially language reception) in patients with migraine. These results were independent of headache history. No significant differences between groups in spatial and motor ability, reading, and arithmetic performances. The association between migraine and verbal functioning impacted on later academic success
Parisi et al. (40)	63 Migraine without aura (25 M; 38 F; mean age 11.0 ± 2.9 years) 19 TTH (9 M; 10 F; mean age 10.9 ± 2.6 years) 79 Control subjects (27 M; 52 F; matched with headache patients)	- General intelligence (WISC-R)	Lower scores in Total and Verbal Intelligence Quotients in patients, especially in children with migraine. No significant differences in the Performance Quotient between the three groups. Total IQ, verbal and performance abilities showed a Significant negative correlation between the Total IQ, Verbal and Performance Quotients and the frequency of attacks. A significant association was found between the age of headache onset and the general intelligence
Esposito et al. (41)	75 Migraine without aura (43 M; 32 F) 72 TTH (49 M; 23 F) 137 Control subjects (93 M; 44 F; mean age 10.78 ± 2.35 years)	- General intelligence (WISC-III)	No difference in Total IQ between the three groups. TTH had a lower Verbal Intelligence Quotient but higher Performance Intelligence Quotient compared with migraine and control subjects
Costa Silva et al. (42)	28 Migraine (6 M; 22 F; age range 10–18 years, mean age 14.39 ± 2.6 years) 26 Control subjects (18 M; 12 F; similar age range, mean age 14.58 ± 2.43 years)	- Memory and learning (Rey Auditory Verbal Learning Test). - Attention- executive functions (Trail Making Test-A and B, and Stroop Test) - Language: Verbal Fluency Test	Patients with migraine displayed lower performance in short and long-term verbal memories and in semantic verbal fluency. Migraine children also had lower scores in visuo-motor tracking, selective attention and speed of processing
Petrusic et al. (43)	44 Migraine with aura (21 M; 23 F; age range 13-19 years; 16.09 ± 2.05 year) 44 Control subjects matched with migraineurs in terms of gender and age	- Attention/orientation, - Memory, - Verbal fluency, - Language, - Visuospatial abilities The neuropsychological evaluation was performed by using the Addenbrooke's Cognitive Examination test	Lower attention/orientation, memory and verbal fluency in patients. No differences between patients and controls in language and visuospatial abilities. Adolescents with more complex aura (higher cortical dysfunctions) showed a poorer cognitive outcome
Haverkamp et al. (44)	37 Migraine (22 M; 15 F; mean age 10 years ± 2.10) 17 Healthy non-affected siblings (6 M; 11 F; mean age 8.81 ± 2.61)	- Mental processing and cognitive development in children (Kaufman Assessment Battery for Children- II)	Patients as well as their siblings demonstrated a neurocognitive performance within the average range. Both patients and their siblings had worse performance in sequential information processing task compared with simultaneous information processing task. No neuropsychological vulnerability in children with aura
Chiappedi et al. (45)	16 Migraine (9 without and 7 with aura) 14 TTH 30 Control subjects Mean age cases: 12.4 years, mean age controls: 12.6 years	- General intelligence abilities (WISC-IV)	No significant differences in the Total IQ score between groups. Patients displayed lower performance in memory skills. A borderline statistical difference between patients and controls in “Verbal Comprehension” Index. No statistically significant differences in cognitive performance according to the headache subtypes, the frequency of attacks and duration of headache
Calandre et al. (32)	10 Migraine with aura and 50 migraine without aura (28 adults and 32 pediatric; age range 15 to 68 years) 30 Control subjects (17M; 13 F)	- General intellectual functions (WAIS) - Attention (Digits backwards -subtest from WAIS; Stroop test; Strub and Black letter list; Trail Making Test- Parts A and B; icture completion- subtest from WAIS) - Memory (Digits forwards- subtest from WAIS; Rey Auditory Verbal Learning Test; Logical memory; Visual reproductio- subtest from the Wechsler Memory Scale; Short and delayed reproduction of Rey-Osterrieth's Complex Figure Test; Benton Visual Retention Test) - Visuo-motor speed processing (Digit Symbol Substitution Test; Visual reaction time - Motor coordination (Luria Sequential Motor Tests; Rapid alternating hand movements; Rhythm test) - Visual perception (Poppelreuter Test; Benton's Recognition Form Test/Visual Form Discrimination; Benton's Facial Recognition Test) - Abstract reasoning. Similarities- subtest from WAIS) - Mental calculation (Arithmetic-subtest from WAIS) - Praxias (Rey's Complex Figure Reproduction; Block design)	Migraine group showed a poor performance only in the visuo-motor speed processing (measured by the reaction time). A significant association between the frequency of attacks and the history of migraine with low attention, visuo-motor speed processing and memory abilities. No relevant difference between patients with and without aura
Riva et al. (46)	14 Migraine with aura (mean age 12.9 ± 2.10) 29 Migraine without aura (11.9 ± 2.8) 19 TTH (12 ± 2.11) 52 Control subjects (11.10 ± 2.1)	- Intellectual abilities (Raven Progressive Matrices) - Sustained attention and response inhibition (Conners' Continuous Performance Test)	Faster mean reaction time, associated with highly activated response and errors, in patients. No relationship between the frequency of attacks, the duration of headache and the attention abilities. No significant neuropsychological differences between the three groups of patients
Villa et al. (47)	25 Patients suffering from migraine without aura. 5 Children with migraine with aura (15 M, 15 F; age range 8–12 years, mean age 10.8 ± 1.5) 30 Control group children (16 M; 14 F; age range 8–12 years, mean age 9.9 ± 1.3)	- Visual attention, mental flexibility, visual scanning, alternate attention and psychomotor velocity (Trail Making Test-A and B; Letter-Canceling Test; Test of Visual Attention)	No difference between patients and control in the reaction time performance. The migraine group had a low performance in all other variables, in particular alternate attention
Iacovelli et al. (25)	16 Migraine without aura (10 M; 6 F; mean age 11.7 ± 2.7 years) 12 TTH (4 M; 8 F; mean age 12.3 ± 2.8) 10 Control subjects (5 M; 5 F; mean age 11.7 ± 2 years	- Visual Attention (Deux Barrage test)	Patients had similar performance in attentive neuropsychological test compared with healthy control. No difference between migraine and TTH was found. A positive correlation between the neurophysiological pattern and the cognitive performance was found only in migraine group
Arruda et al. (48)	510 Episodic migraine (242 M; 268 F) 36 Chronic migraine (18 M; 18 F) 726 Episodic TTH (369 M; 357 F) 1 Chronic TTH (1 M; 0 F) (age range 5–12 years)	- ADHD (Multimodal Treatment Study for Attention-Deficit/Hyperactivity Disorder- IV and Strengths and Difficulties questionnaires performed by parents and teachers)	The prevalence of ADHD was significantly higher in children with migraine overall. An association between ADHD symptoms (in particular inattentive) and headache frequency
Attygalle et al. (49)	141 Migraine (mean age 10.6 ± 2.4 years; 56.8% females) 85 Control subjects (9.2 ± 2.8 years; 38.1% females) (age range 5–14 years)	- ADHD (the abbreviated version of the Swanson, Nolan, and Pelham (SNAP) Questionnaire (SNAP-IV)	Higher percentage of ADHD inattention and impulsive-hyperactive symptoms in patients with migraine
Genizi et al. (50)	279 High-school students (158 M; 121 F; age range 15–16 years) of whom 230 reported headache	A computerized general health questionnaire	27% of students who reported headache had a diagnosis of ADHD
Pavone et al. (51)	56 Migraine 224 TTH 280 Control subjects (age range 4–14 years)	Parents were requested to answer questions regarding of ADHD symptoms	No difference in ADHD symptoms between groups
Genizi et al. (52)	107 Migraine 116 TTH (108 M; 135 F; age range 6–18 years; mean age 11.24 ± 3.08)	- ADHD (Conners' Parents and Teacher Rating Scales-Revised) - Parents and patients evaluated school performance	Among patients with headache, 24,7% were diagnosed with learning disabilities and 28% with ADHD. Children with recurrent attacks (>10 in a month) and long headache duration were more prone to learning disabilities. ADHD and poor school academic performance were less prevalent among patients with migraine compared with TTH patients. Migraine showed a higher prevalence of learning disabilities
Genizi et al. (50)	262 Migraine (124 M; 138 F; mean age 12 ± 2.9 years): 68 Migraine with aura 59 Children met the criteria for episodic TTH/mixed headaches in addition to migraine headaches	- General intelligence (WISC- III)	Children with mixed headaches were more likely to have a learning disability than children with migraine alone
Moutran et al. (53)	30 Migraine (15 M; 15 F; age range 8–12 years, mean age 10.8 ± 1.6 years) 30 Control subjects (18 M; 12 F; similar age range, mean age 10.0 ± 1.3 years)	- General intelligence (WISC-III)	Patients had normal intelligence performance. Migraine group displayed a significant lower score on vocabulary, information, arithmetic and object assembly subtests. Patients showed significant lower performance in perceptual organization, resistance to distraction, and processing speed indexes
Esposito et al. (54)	27 Migraine without aura (11 M; 16 F) (mean age: 8.7 ± 2.1 years) 59 Control subjects (34 M; 25 F) (mean age: 8.0 ± 2.1 years)	- General intelligence (WISC-III) - Visual-motor integration skills (Beery visual-motor integration test, VMI)	Low performance in visual motor integration abilities and motor coordination skills in patients suffering from migraine without aura
Esposito et al. (55)	71 Migraine without aura (39 M; 32 F females; mean age: 9.13 ± 1.94 years) 93 Control subjects (44 females, 49 males; mean age: 8.97 ± 2.03 years)	- Visual-motor integration skills (Beery visual-motor integration test, VMI) - Motor coordination performance (Movement Assessment Battery for Children, M-ABC) - Nintendo Wii Fit Plus™ system as a rehabilitative device	Low performance in visual-motor integration abilities and motor coordination skills in patients suffering from migraine without aura. Positive effects of the Nintendo Wii Fit PlusTM system as a rehabilitative device
Precenzano et al. (56)	84 Children with migraine without aura (45 M; 39 F; mean age: 8.91 ± 2.46 years), randomly divided into two groups	- Motor coordination (VMI) - Software “Allenare le abilitsuo-spaziali” for strengthening of non-verbal and skills visual-spatial	Efficacy of the software training

*CMP, colored progressive matrices; WISC-R, Wechsler intelligence scale for children-revised; WISC-III, Wechsler intelligence scale for children - third edition; WISC-IV, Wechsler intelligence scale for children - fourth edition; ADHD, attention deficit hyperactive disorder*.

While results of studies evaluating non-verbal intelligence agree in showing normal values in children and adolescent with migraine, findings obtained from global intelligence tests show a peculiar profile characterized by subclinical difficulties in verbal skills (14, 34, 40, 45). Results, however, are largely heterogeneous (Table 1). Waldie et al. explored the cognitive performances of headache patients from the age of 3–26 years (34). To the best of our knowledge, the authors were the first to evaluate the general intellectual ability of children and adolescents with migraine and TTH, focusing on different and specific cognitive domains (verbal and performance abilities). Using the “Wechsler Intelligence Scale for Children-Revised” (WISC-R) (58) at ages of 7, 9, 11, and 13 years, the authors observed lower verbal skills (in particular verbal comprehension) in patients with migraine compared not only with free control subjects (Verbal-IQ mean score = 100.6 ± 11.4 and 105.7 ± 13.5, respectively), but also with patients with TTH (mean score = 104.4 ± 13.8). Low verbal receptive performances were also found in 3 years old migraine patients tested with “Peabody Picture Vocabulary test” (PPVT) (59), which evidenced a strong correlation with the mean WISC Verbal-IQ (*p* < 0.001). Data analysis showed that the verbal performance in patients with migraine did not decline with age and remained lower than other groups over the time. On the other hand, verbal expression measured by the “Illinois Test of Psycholinguistic Ability” (ITPA) (60) did not differ between the headache groups. The cognitive abilities were not related to duration of disease, use of medications or duration and severity of migraine attacks. These findings led the authors to suppose that the low verbal abilities evidenced in migraine group could not be imputable to a cause-effects relationship between history of migraine and cognitive performance, but was rather due to a “not-identified” common risk factor presumably occurring in a prenatal developmental phase. As alternative hypothesis, the authors suggested that the lower performances could be due to patients' attention and concentration difficulties (34).

The absence of association between the intelligence profile and migraine features in children and adolescents was also confirmed in a more recent study (45). The authors described the risk of lower verbal performance in young patients with headache diagnosis (20 children with migraine and 10 with TTH, age ranging from 11 to 14 years) in comparison with healthy children. Patients' group showed a normal TIQ (mean = 112.80 ± 13.4), but exhibited a characteristic neuropsychological functioning regarding memory and verbal skills. The authors identified lower scores in the “Working memory” and, though not statistically significant, in “Verbal Comprehension” indexes of WISC-IV test (61). Duration of headache and frequency of the attacks did not influence the results. Moreover, patients with migraine and TTH had a similar cognitive profile.

In pediatric patients with headache, lower resources in verbal skills and in total intelligence were also reported by Parisi et al. (40) who compared the functioning of children diagnosed with migraine without aura (n. 63) and TTH (n.19) with that of a control healthy group (n. 79) by using the WISC-R edition (58). The authors found lower total quotient (TIQ) scores in children with migraine than in control subjects, although no difference between headache groups was found. Moreover, the study confirmed the lower performance in verbal quotient (VIQ), in particular comprehension subscale, in children with migraine and TTH, as compared to control group. On the other hand, no difference between groups was found in the performance scale (PIQ). In disagreement with the previous study by Waldie et al. (34) the authors showed a significant negative correlation between the frequency of attacks and total, verbal and performance intelligence quotients of WISC test and between the age of headache onset and the total intelligence quotient score. It was suggested that cognitive impairment in headache, particularly migraine, may be due to repeated hyperactivation of several cortical or subcortical neuronal networks during the attacks.

A different intelligence profile between migraine and TTH patients was described by Esposito et al. (41). The authors confirmed a normal intelligence development in patients with primary headache, although, in disagreement with previous literature, they showed weak perceptual organization competences in patients with migraine as compared with both TTH patients and healthy controls. Abnormalities in frontal lobe structure and impairment of executive functions have been suggested as causal mechanisms.

Studies focusing on receptive and expressive language in young patients with headache are sparse. To the best of our knowledge, in addition to the above mentioned study by Waldie et al. (34), only two other studies examined specific interictal language abilities in children/adolescents with migraine. In 2016, Costa Silva et al. evaluated semantic and phonological fluency (“Verbal Fluency test”) in migraine patients (n. 28) and healthy children (n. 26) (42). In patients with migraine, there were significant lower scores in semantic verbal fluency (“animals” subtest), but not in phonemic verbal fluency. Petrusic et al. (43) recruited 74 adolescents (age range 13–19 years) who suffered from migraine with aura. Compared with healthy subjects, performances in verbal fluency, but not in language, were lower in patients than in control subjects. The study did not show a significant association between the neuropsychological performance, including language and verbal fluency, and both higher cortical dysfunctions and aura frequency/duration.

### Memory

Several studies investigated memory abilities in children and adolescents with migraine by using standardized test (Table 1). In an early study, D'Andrea et al. (37) described impaired short and long-term visuo-spatial (“Rey Memory test”) and verbal (“Logical Memory test,” the “t, and 10 word learning test”) memory. Several years later, by using the Kaufman Assessment Battery for Children (K-ABC) (62), Haverkamp et al. (44) found cognitive performance within the normal range in children with migraine (n. 37, mean age = 10 years) and in their siblings (n.17, mean age = 8.81 years). The authors, however, described lower performance in subtests requiring memory competences, as in sequential information processing tasks (SEQ). No neuropsychological vulnerability in patients with aura was revealed. Considering that similar scores were found in both migraine patients and their sibling, the authors hypothesized a sample artifact and concluded that no general risk for defective cognitive development exists in young patients with migraine. Using the structured test “Rey Auditory Verbal Learning test” (RAVLT) (63), deficits in verbal memory and learning were reported (42). Compared with healthy subjects, migraine patient performance was more influenced by distractors. Difficulties in verbal memory and, in particular, registration, consolidation, and recall of verbal stimuli were found. As discussed by the authors, since these functions are associated with the ability to use schemas to seek information and organize thoughts, their impairment may lead to poor school performance. The results of a study by Chiappedi et al. (45) provided confirmatory evidence that adolescents affected by headache (migraine and TTH) may have short-term memory difficulties. Although a specific structured evaluation of memory was not performed, cognitive test WISC-IV showed specific impairment in the “Working Memory” index, especially concerning the subtest “Digit Span,” which provides a measure of the ability to store, manage and manipulate information indispensable for language and learning. The study failed to find statistically significant differences in working memory abilities according to headache diagnosis, frequency of attacks, and duration of headache.

In disagreement with previous studies, Margari et al. (39) reported the absence of significant differences between patients with primary headache and controls in “Forward Memory,” “Reverse Memory” and “Non-verbal Memory” subtests scores, issued from the cognitive non-verbal test “Leiter-3” (57). However, the study described a significant association of frequency of the attacks and headache disability with non-verbal memory abilities, suggesting that higher headache disability may have a significant impact on specific cognitive domains, such as the non-verbal memory.

Focusing on migraine with aura, Petrusic et al. (43) showed significantly lower performance in memory skills in adolescents suffering from migraine with aura compared with healthy subjects. As for language, the authors did not evidence significant association between the presence of higher cortical dysfunctions, frequency and duration of aura and the memory skills (evaluated by the “ACE-R test”) (43, 64).

Data on the role of migraine features on memory abilities in pediatric patients are sparse (Table 1). To the best of our knowledge, in addition to the above mentioned studies (39, 45), only Calandre et al. (32) explored this topic. In a sample of 28 adults and 32 pediatric patients with migraine, the authors described an impairment of short- and long-term visual memory, verbal memory, and working memory, related to frequency of the attacks and headache history. Multiple hypoperfusion areas demonstrated by Single Photon Emission Computed Tomography (SPECT) suggested an involvement of several cerebral areas in both hemispheres.

### Attention and Attention Deficit Hyperactive Disorder

Research suggests difficulties in attention, processing speed and executive functions during the interictal period of migraine (14) (Table 1). Riva et al. (38) investigated the cognitive performances and behavioral problems in children and adolescents affected by migraine with and without aura (17 and 31 patients, respectively). Normal visual, selective and divided, attention was found in both groups of patients, evaluated with a paper-and-pencil test (“Trail making test,” TMT A and B) (65). However, slower information processing speed, assessed by a computerized simple reaction time to visual stimuli task, was found in migraine patients compared to controls. Frequency of the attacks, but not the duration of the disease, influenced the simple reaction time (38). Confirmatory evidence of altered reaction time in young headache patients was provided by a later study of the same authors (46). The “Conners' Continuous Performance test” (66), evaluating selective and sustained attention, was administered to 62 young patients with primary headache (14 with migraine with aura, 29 with migraine without aura and 19 with TTH) and 52 controls. Faster mean reaction time, associated with highly activated response and errors (more frequent false positive responses), was found in patients compared with controls, suggesting an impulsive response style. The role of emotional factors, such as anxiety, in attentive performance of patients was hypothesized. As in their previous study, no difference was found between the groups of patients, suggesting that migraine and TTH may represent a continuum and share the same pathophysiological mechanisms (22, 24). In a more recent study (42), low performance in attention and executive functions tests was described in children with migraine. In particular, a significant longer execution time (“Trail-Making test”) was found in migraine patients compared with healthy subjects. On the other hand, patients and controls exhibited similar performances in errors. Considering that reaction time explores mainly the speed in detecting and responding to a signal, the authors suggested that its abnormality can be considered as an early and subclinical sign of a neuropsychological impairment, associated with the frequency of headache attacks. In contrast with these findings, another study (44) described similar sequential and simultaneous information processing abilities (evaluated by Kaufman-Assessment Battery for Children) (62) in patients with migraine (with and without aura) and their healthy siblings. These results were replicated by Villa et al. (47), who found no differences between migraine patients (n. 30) and healthy children (n. 30) in a visual reaction time task (“Visual attention test”) (47, 67).

Peculiar attention mechanisms in young patients with migraine were suggested by psychophysiological research (22). Agessi et al. (68) showed impairment of auditory selective attention and physiologic mechanisms of temporal processing. In 2011, we explored both neuropsychological and neurophysiological components of attention in children with migraine (n. 16), TTH (n. 12), and healthy subjects (n. 10) (25). Although a paper and pencil attentive test (“Deux Barrage”) (25) did not evidence any significant impairments and differences in speed and errors between groups, the authors found abnormal neurophysiological mechanisms of spatial attention in patients with headache. Moreover, only in migraine patients, and not in those with TTH, the N140 amplitude increase showed a positive correlation with the neuropsychological performance. We hypothesized that in children with migraine normal attention achievement can be obtained involving a larger amount of “frontal lobe resources,” as compared to healthy children (25).

Though a causal association cannot be maintained, a high prevalence of ADHD symptoms has been described in children and adolescents suffering from migraine (48, 49, 69) (Table 1). Arruda et al. (48) showed a significant higher prevalence of ADHD in children with migraine compared with control subjects. In particular, since this comorbidity mostly involved children with chronic migraine, it was suggested that frequency of migraine attacks increased the risk of ADHD. These results were confirmed by Attygalle et al. (49). The authors described a higher percentage of inattention and impulsive-hyperactive symptoms in patients with migraine than in control subjects. Exploring the opposite association, a high prevalence of migraine (and other primary headache) was described in children with ADHD (70).

We must underline that the association between pediatric headache, in particular migraine, and ADHD is not generally accepted. In a cohort study conducted on 279 high-school students, ADHD prevalence was not different between students who reported headache and those who did not (50). These results were in accordance with a previous study of Pavone et al. (51). In order to explore the prevalence of ADHD and learning disabilities in pediatric patients with headache, a total of 243 medical records of patients (6–18 years) with migraine (n. 107) and TTH (n. 116) were retrospectively reviewed by Genizi et al. (52). Higher rates of ADHD, but also learning disabilities, were found in headache children than in the general population. However, the study did not confirm the relationship between ADHD symptoms and migraine. Supporting a previous study of Mazzone et al. (71), the research revealed a higher prevalence of ADHD symptoms and lower school achievements in children and adolescents with TTH compared to those with migraine.

### Learning Disabilities

Learning disabilities are a heterogeneous group of disorders involving difficulties mainly in reading, writing, or mathematical abilities (Diagnostic and Statistical Manual of Mental Disorders-Fifth Edition, DSM-5) (72). These disorders show very often a comorbidity with impairments in cognitive domains, such as memory, attention and processing speed. Few studies investigated the association between migraine and learning disabilities (Table 1). While some of them reported normal abilities in arithmetic and reading in children with migraine (34, 37), other authors described an association between learning disabilities and migraine. Genizi (52) found that about 25% of patients referred to a headache center reported learning disabilities. In particular, compared with children with TTH, those with migraine showed a higher prevalence of learning disabilities (respectively, 26.4 vs. 21.7%). Children with more than 10 attacks/month and long headache duration were more prone to show learning disabilities. Despite these results, children with migraine had good to excellent school achievement. The authors hypothesized that fear of failure and desire for successes, typical of children with headache, may lead to an approach to school characterized by over achievement. Some years later, the same authors (50) showed a higher prevalence of learning disabilities in children with mixed headache (migraine and TTH) compared to those with migraine alone. We have to underline that most studies did not provide a specific neuropsychological evaluation for learning disabilities.

### Visuo-Motor and Visuo-Spatial Abilities

Data on visuo-motor and visuo-spatial abilities of children with migraine during the interictal phase are sparse (Table 1). In a study by Correa Moutran et al., a low perceptual organization (evidenced by WISC-III) was observed (53, 73). On the other hand, no difference in visuospatial skills between migraneurs with aura and controls was found in a later study using “Addenbrooke's Cognitive Examination” test (43). In 2012, Esposito et al. showed a lower performance in motor coordination and visual motor integration evaluation in children with migraine, as compared with controls (54). Using the “Beery Visual-motor integration” test (VMI) (74) and the “Movement Assessment Battery” for Children (M-ABC) (75), the same authors confirmed a low performance in visual motor integration abilities and motor coordination skills in patients suffering from migraine without aura (55). Effectiveness of the Nintendo Wii Fit Plus™ system as a rehabilitative device in these patients was suggested (55). Also, Precenzano et al. showed the efficacy of a specific software training (“Allenare le abilitsuo-spaziali”) for enhancing the visuospatial abilities (56).

## Discussion

The reviewed literature suggests that, though considered a benign disease, pediatric primary headache may also be associated to altered neuropsychological functioning in the interictal phase. Available findings provide evidence of the role of cognitive difficulties in poor academic achievement in children with headache (34, 40).

Although migraine was especially investigated, literature data suggest that some cognitive abilities, such as memory and attention, may be impaired also in children with TTH. These results support the hypothesis that, in pediatric age, migraine and TTH are not distinct entities, but two aspects of the same spectrum of benign headache (22, 24, 46).

The interictal cognitive profile of pediatric patients with primary headache is characterized also by dysfunctions in selective, prolonged and alternate attention. It was shown that pediatric patients with migraine and TTH have altered processing speed and reaction time, suggesting an impulsive response style and impaired executive functioning. Although children and adolescents with migraine generally show a normal intelligence, they may have difficulties in verbal skills, in particular comprehension abilities. Some authors hypothesized that this may be related to a risk factor, common to migraine and language, occurring in an early developmental, even prenatal, phase (34).

Data on vulnerability factors leading to cognitive impairment in children with primary headache are sparse. The mechanisms underlying the relationship between migraine and interictal cognitive impairment are not completely known. Shared neurobiological factors, such as neurotransmitters, play a key role in both migraine and cognition (76). Noradrenaline and dopamine are involved in memory, learning and attention, as well in several components of executive functions. While noradrenaline keeps adequate levels of alternate, sustained, and selective attention, dopaminergic connections regulate cognitive functions associated with working memory and attention skills. Dysfunction of the hypothalamic-pituitary-adrenal axis (77) and dopamine networks (78) have been suggested as common factors underlying migraine and ADHD. Both noradrenaline and dopamine are involved also in migraine pathophysiology, pain generation as well as accompanying symptoms of migraine attacks (79, 80). Noradrenaline and dopamine act on the trigeminal system by modulating neuronal excitability (81, 82). Low levels of noradrenaline may predispose patients with migraine to syncope and headache attacks. On the other hand, several prodromal symptoms (mood changes, somnolence), accompanying symptoms (vomiting and nausea), and postdromal symptoms (tiredness, drowsiness, mood changes) of the migraine attack may be related to dopaminergic activation (80).

An increasing body of research explored the role of pediatric migraine severity on cognitive resources in the headache free period (32, 38–40, 45, 46, 82). There is evidence that early age of onset and high frequency of the attacks may represent factors favoring cognitive impairment (32, 40).

Though out of the purpose of the present review, the role of medications in causing cognitive difficulties in children with primary headache is not to be forgotten. A negative influence, as possible side-effects of drugs, has been described by Powers et al. (83), who reported memory impairment (17%) and a not specified “cognitive disorder” (16%) in subjects with migraine treated with topiramate. However, the relationship between pharmacological treatment may be bi-univocal. Villa demonstrated lower performance, although within the normal range, in a visual selective and alternate attention test in untreated migraine patients compared with both children assuming migraine prophylaxis and control subjects (84). The authors supposed a positive influence of prophylactic treatment in re-establishing the equilibrium of neurotransmitters and, as a consequence, a reinforcement of the attentive functions (84).

Reviewed literature shows several limitations and heterogeneity in the used methods. Firstly, most recruited patients are referred to neurologists or specialistic headache clinics, resulting unrepresentative of the general population. Second, included samples can be very small and do not consider the migraine phenotype, such as migraine with or without aura. Third, some studies lack a control group. Forth, the neuropsychological tools used for patient evaluation are extremely variable. Fifth, in some studies neuropsychological information are only issued from parents' and/or teachers' reports. Lastly, the possible role of psychological symptoms in explaining, at least in part, the association between pediatric migraine and cognitive difficulties has been scarcely investigated (38, 82).

## Conclusions

Pediatric migraine may be associated with altered neuropsychological functioning involving language, attentional resources, processing speed and memory, particularly verbal memory. Given the impact that this disease can have on school performance and the tendency to persist from childhood to adulthood, a special attention should be paid to patients' cognitive development. Unfortunately, the heterogeneity of the results obtained by the different neurocognitive tests prevents statistical analysis of the published findings to be performed. Additional neuropsychological research evaluating larger samples and using more homogenous methods is needed.

## Author Contributions

ST and MPC: conceptualization. ST and MV: writing—original draft preparation. MPC, LP, FU, MANF, RM, GS, and GM: writing—review and editing. MV, TGC, and FV: supervision. All authors have read and agreed to the published version of the manuscript.

## Conflict of Interest

The authors declare that the research was conducted in the absence of any commercial or financial relationships that could be construed as a potential conflict of interest.

## Publisher's Note

All claims expressed in this article are solely those of the authors and do not necessarily represent those of their affiliated organizations, or those of the publisher, the editors and the reviewers. Any product that may be evaluated in this article, or claim that may be made by its manufacturer, is not guaranteed or endorsed by the publisher.

## References

[1] Wöber-Bingöl C. Epidemiology of migraine and headache in children and adolescents. Curr Pain Headache Rep. (2013) 17:341. 10.1007/s11916-013-0341-z 23700075

[2] Krogh A-B Larsson B Linde M. Prevalence and disability of headache among Norwegian adolescents: a cross-sectional school-based study. Cephalalgia Int J Headache. (2015) 35:1181–91. 10.1177/0333102415573512 25720767

[3] Headache Classification Committee of the International Headache Society (IHS). The international classification of headache disorders, 3rd edition (beta version). Cephalalgia Int J Headache. (2013) 33:629–808. 10.1177/0333102413485658 23771276

[4] Leviton A. Do learning handicaps and headache cluster? J Child Neurol. (1986) 1:372–7. 10.1177/088307388600100411 3598136

[5] Pawlowski C Buckman C Tumin D Smith AW Crotty J. National trends in pediatric headache and associated functional limitations. Clin Pediatr. (2019) 58:1502–8. 10.1177/0009922819875560 31522542

[6] Arruda MA Bigal ME. Migraine and migraine subtypes in preadolescent children: association with school performance. Neurology. (2012) 79:1881–8. 10.1212/WNL.0b013e318271f812 23109652

[7] Rocha-Filho PAS Santos PV. Headaches, quality of life, and academic performance in schoolchildren and adolescents. Headache. (2014) 54:1194–202. 10.1111/head.12394 24898739

[8] Powers SW Patton SR Hommel KA Hershey AD. Quality of life in paediatric migraine: characterization of age-related effects using PedsQL 4.0. Cephalalgia Int J Headache. (2004) 24:120–7. 10.1111/j.1468-2982.2004.00652.x 14728707

[9] Varni JW Limbers CA Burwinkle TM. Impaired health-related quality of life in children and adolescents with chronic conditions: a comparative analysis of 10 disease clusters and 33 disease categories/severities utilizing the PedsQL 4.0 generic core scales. Health Qual Life Outcomes. (2007) 5:43. 10.1186/1477-7525-5-43 17634123 PMC1964786

[10] Powers SW Patton SR Hommel KA Hershey AD. Quality of life in childhood migraines: clinical impact and comparison to other chronic illnesses. Pediatrics. (2003) 112:e1–5. 10.1542/peds.112.1.e1 12837897

[11] Breuner CC Smith MS Womack WM. Factors related to school absenteeism in adolescents with recurrent headache. Headache. (2004) 44:217–22. 10.1111/j.1526-4610.2004.04050.x 15012658

[12] Gorodzinsky AY Hainsworth KR Weisman SJ. School functioning and chronic pain: a review of methods and measures. J Pediatr Psychol. (2011) 36:991–1002. 10.1093/jpepsy/jsr038 21745810

[13] Strine TW Okoro CA McGuire LC Balluz LS. The associations among childhood headaches, emotional and behavioral difficulties, and health care use. Pediatrics. (2006) 117:1728–35. 10.1542/peds.2005-1024 16651331

[14] Termine C Bartoli B Agosti MA Cavanna AE Balottin U. Cognitive impairment in children and adolescents with migraine. Front Neurol. (2018) 9:667. 10.3389/fneur.2018.00667 30154756 PMC6102948

[15] Vincent MB Hadjikhani N. Migraine aura and related phenomena: beyond scotomata and scintillations. Cephalalgia Int J Headache. (2007) 27:1368–77. 10.1111/j.1468-2982.2007.01388.x 17944958 PMC3761083

[16] Martins IP. Cognitive performance in chronic migraine. Arq Neuropsiquiatr. (2020) 78:131–2. 10.1590/0004-282x20200024 32348417

[17] Gil-Gouveia R Oliveira AG Martins IP. Subjective cognitive symptoms during a migraine attack: a prospective study of a clinic-based sample. Pain Physician. (2016) 19:E137–50. 26752482

[18] Gil-Gouveia R Martins IP. Clinical description of attack-related cognitive symptoms in migraine: a systematic review. Cephalalgia Int J Headache. (2018) 38:1335–50. 10.1177/0333102417728250 28847155

[19] Quintela E Castillo J Muñoz P Pascual J. Premonitory and resolution symptoms in migraine: a prospective study in 100 unselected patients. Cephalalgia Int J Headache. (2006) 26:1051–60. 10.1111/j.1468-2982.2006.01157.x 16919055

[20] Evers S Quibeldey F Grotemeyer KH Suhr B Husstedt IW. Dynamic changes of cognitive habituation and serotonin metabolism during the migraine interval. Cephalalgia Int J Headache. (1999) 19:485–91. 10.1046/j.1468-2982.1999.019005485.x 10403063

[21] Cavestri R Arreghini M Longhini M Ferrarini F Gori D Ubbiali A. Interictal abnormalities of regional cerebral blood flow in migraine with and without aura. Minerva Med. (1995) 86:257–64. 7566559

[22] Pro S Tarantino S Capuano A Vigevano F Valeriani M. Primary headache pathophysiology in children: the contribution of clinical neurophysiology. Clin Neurophysiol Off J Int Fed Clin Neurophysiol. (2014) 125:6–12. 10.1016/j.clinph.2013.04.335 23756059

[23] Schoenen J. Neurophysiological features of the migrainous brain. Neurol Sci Off J Ital Neurol Soc Ital Soc Clin Neurophysiol. (2006) 27(Suppl. 2):S77–81. 10.1007/s10072-006-0575-1 16688634

[24] Valeriani M Galli F Tarantino S Graceffa D Pignata E Miliucci R. Correlation between abnormal brain excitability and emotional symptomatology in paediatric migraine. Cephalalgia Int J Headache. (2009) 29:204–13. 10.1111/j.1468-2982.2008.01708.x 18823365

[25] Iacovelli E Tarantino S De Ranieri C Vollono C Galli F De Luca M. Psychophysiological mechanisms underlying spatial attention in children with primary headache. Brain Dev. (2012) 34:640–7. 10.1016/j.braindev.2011.10.005 22099868

[26] De Tommaso M Valeriani M Guido M Libro G Specchio LM Tonali P. Abnormal brain processing of cutaneous pain in patients with chronic migraine. Pain. (2003) 101:25–32. 10.1016/s0304-3959(02)00299-3 12507697

[27] Rocca MA Messina R Colombo B Falini A Comi G Filippi M. Structural brain MRI abnormalities in pediatric patients with migraine. J Neurol. (2014) 261:350–7. 10.1007/s00415-013-7201-y 24305994

[28] Humbertclaude V Riant F Krams B Zimmermann V Nagot N Annequin D. Cognitive impairment in children with CACNA1A mutations. Dev Med Child Neurol. (2020) 62:330–7. 10.1111/dmcn.14261 31115040

[29] Messina R Rocca MA Colombo B Valsasina P Horsfield MA Copetti M. Cortical abnormalities in patients with migraine: a surface-based analysis. Radiology. (2013) 268:170–80. 10.1148/radiol.13122004 23533286

[30] Guarnera A Bottino F Napolitano A Sforza G Cappa M Chioma L. Early alterations of cortical thickness and gyrification in migraine without aura: a retrospective MRI study in pediatric patients. J Headache Pain. (2021) 22:79. 10.1186/s10194-021-01290-y 34294048 PMC8296718

[31] Schmitz N Arkink EB Mulder M Rubia K Admiraal-Behloul F Schoonman GG. Frontal lobe structure and executive function in migraine patients. Neurosci Lett. (2008) 440:92–6. 10.1016/j.neulet.2008.05.033 18556120

[32] Calandre EP Bembibre J Arnedo ML Becerra D. Cognitive disturbances and regional cerebral blood flow abnormalities in migraine patients: their relationship with the clinical manifestations of the illness. Cephalalgia Int J Headache. (2002) 22:291–302. 10.1046/j.1468-2982.2002.00370.x 12100092

[33] Mathew NT Meyer JS Welsch KM Neblett CR. Abnormal CT-Scans in migraine. Headache. (1977) 16:272–9. 10.1111/j.1526-4610.1976.hed1606272.x 830616

[34] Waldie KE Hausmann M Milne BJ Poulton R. Migraine and cognitive function: a life-course study. Neurology. (2002) 59:904–8. 10.1212/wnl.59.6.904 12297575

[35] Foti M Lo Buono V Corallo F Palmeri R Bramanti P Marino S. Neuropsychological assessment in migraine patients: a descriptive review on cognitive implications. Neurol Sci Off J Ital Neurol Soc Ital Soc Clin Neurophysiol. (2017) 38:553–62. 10.1007/s10072-017-2814-z 28101762

[36] De Araújo CM Barbosa IG Lemos SMA Domingues RB Teixeira AL. Cognitive impairment in migraine: a systematic review. Dement Neuropsychol. (2012) 6:74–9. 10.1590/S1980-57642012DN06020002 29213777 PMC5619244

[37] D'Andrea G Nertempi P Ferro Milone F Joseph R Cananzi AR. Personality and memory in childhood migraine. Cephalalgia Int J Headache. (1989) 9:25–8. 2706672 10.1046/j.1468-2982.1989.0901025.x

[38] Riva D Aggio F Vago C Nichelli F Andreucci E Paruta N. Cognitive and behavioural effects of migraine in childhood and adolescence. Cephalalgia Int J Headache. (2006) 26:596–603. 10.1111/j.1468-2982.2006.01072.x 16674769

[39] Margari L Palumbi R Lecce PA Craig F Simone M Margari M. Non-verbal cognitive abilities in children and adolescents affected by migraine and tension-type headache: an observational study using the leiter-3. Front Neurol. (2018) 9:78. 10.3389/fneur.2018.00078 29556207 PMC5844943

[40] Parisi P Verrotti A Paolino MC Urbano A Bernabucci M Castaldo R. Headache and cognitive profile in children: a cross-sectional controlled study. J Headache Pain. (2010) 11:45–51. 10.1007/s10194-009-0165-8 19841863 PMC3452186

[41] Esposito M Pascotto A Gallai B Parisi L Roccella M Marotta R. Can headache impair intellectual abilities in children? An observational study. Neuropsychiatr Dis Treat. (2012) 8:509–13. 10.2147/NDT.S36863 23139628 PMC3490685

[42] Costa-Silva MA Prado AC de A de Souza LC Gomez RS Teixeira AL. Cognitive functioning in adolescents with migraine. Dement Neuropsychol. (2016) 10:47–51. 10.1590/S1980-57642016DN10100009 29213431 PMC5674914

[43] Petrusic I Pavlovski V Savkovic Z Vucinic D Filipovic B Jancic J. Addenbrooke's cognitive examination test for brief cognitive assessment of adolescents suffering from migraine with aura. Acta Neurol Belg. (2017) 117:97–102. 10.1007/s13760-016-0655-9 27255917

[44] Haverkamp F Hönscheid A Müller-Sinik K. Cognitive development in children with migraine and their healthy unaffected siblings. Headache. (2002) 42:776–9. 10.1046/j.1526-4610.2002.02179.x 12390640

[45] Chiappedi M Mensi M Antonaci E Zavani E Tronconi L Termine C. Intellectual profile of adolescents with headache: a case-control study using the WISC-IV. Front Neurol. (2018) 9:128. 10.3389/fneur.2018.00128 29559952 PMC5845748

[46] Riva D Usilla A Aggio F Vago C Treccani C Bulgheroni S. Attention in children and adolescents with headache. Headache. (2012) 52:374–84. 10.1111/j.1526-4610.2011.02033.x 22085321

[47] Villa TR Correa Moutran AR Sobirai Diaz LA Pereira Pinto MM Carvalho FA Gabbai AA. Visual attention in children with migraine: a controlled comparative study. Cephalalgia Int J Headache. (2009) 29:631–4. 10.1111/j.1468-2982.2008.01767.x 19187339

[48] Arruda MA Arruda R Guidetti V Bigal ME. ADHD is comorbid to migraine in childhood: a population-based study. J Atten Disord. (2020) 24:990–1001. 10.1177/1087054717710767 28587507

[49] Attygalle UR Hewawitharana G Wijesinghe CJ. Migraine, attention deficit hyperactivity disorder and screen time in children attending a Sri Lankan tertiary care facility: are they associated? BMC Neurol. (2020) 20:275. 10.1186/s12883-020-01855-5 32640997 PMC7341609

[50] Genizi J Khourieh Matar A Schertz M Zelnik N Srugo I. Pediatric mixed headache -The relationship between migraine, tension-type headache and learning disabilities - in a clinic-based sample. J Headache Pain. (2016) 17:42. 10.1186/s10194-016-0625-x 27102119 PMC4840135

[51] Pavone P Rizzo R Conti I Verrotti A Mistretta A Falsaperla R. Primary headaches in children: clinical findings on the association with other conditions. Int J Immunopathol Pharmacol. (2012) 25:1083–91. 10.1177/039463201202500425 23298498

[52] Genizi J Gordon S Kerem NC Srugo I Shahar E Ravid S. Primary headaches, attention deficit disorder and learning disabilities in children and adolescents. J Headache Pain. (2013) 14:54. 10.1186/1129-2377-14-54 23806023 PMC3698063

[53] Moutran ARC Villa TR Diaz LAS Noffs MH da S Pinto MMP Gabbai AA. Migraine and cognition in children: a controlled study. Arq Neuropsiquiatr. (2011) 69:192–5. 10.1590/s0004-282x2011000200010 21537559

[54] Esposito M Verrotti A Gimigliano F Ruberto M Agostinelli S Scuccimarra G. Motor coordination impairment and migraine in children: a new comorbidity? Eur J Pediatr. (2012) 171:1599–604. 10.1007/s00431-012-1759-8 22673929

[55] Esposito M Ruberto M Gimigliano F Marotta R Gallai B Parisi L. Effectiveness and safety of Nintendo Wii Fit PlusTM training in children with migraine without aura: a preliminary study. Neuropsychiatr Dis Treat. (2013) 9:1803–10. 10.2147/NDT.S53853 24453490 PMC3890965

[56] Precenzano F Ruberto M Parisi L Salerno M Maltese A Gallai B. Visual-spatial training efficacy in children affected by migraine without aura: a multicenter study. Neuropsychiatr Dis Treat. (2017) 13:253–8. 10.2147/NDT.S119648 28184165 PMC5291325

[57] Cornoldi C Giofrè D Bellacchi C. Procedure di Somministrazione E Scoring Dei Subtest. Leiter-3 Leiter International Scale. Firenze: Giunti OS Organizzazioni Speciali srl Adattamento italiano (2016).

[58] Petrosko J. Wechsler intelligence scale for children—revised, 1974. David Wechsler. Meas Eval Guid. (1975) 7:265–7.

[59] Taylor Lorne J. The peabody picture vocabulary test: what does it measure? Perceptual and Motor Skills. (1975) 41:777–8.

[60] Kirk SA Winifred D Kirk James J. Illinois Test of Psycholinguistic Abilities. McCarthy: University of illinois press (1968).

[61] Wechsler D. Wechsler Intelligence Scale For Children–Fourth Edition (WISC-IV). San Antonio, TX: The Psychological Corporation (2003).

[62] Melchers P Ulrich P. Kaufman Assessment Battery for Children: K-ABC; Deutschsprachige Fassung. Amsterdam: Swets und Zeitlinger (1994).

[63] Rey A. L'examen psychologique dans les cas d'encéphalopathie traumatique. Arch Psychol. (1941) 28:286–340.16987634

[64] Mioshi E Dawson K Mitchell J Arnold R Hodges JR. The addenbrooke's cognitive examination revised (ACE-R): a brief cognitive test battery for dementia screening. Int J Geriatr Psychiatry. (2006) 21:1078–85. 10.1002/gps.1610 16977673

[65] Reitan RM. Trail making test results for normal and brain-damaged children. Percept Mot Skills. (1971) 33:575–81. 10.2466/pms.1971.33.2.575 5124116

[66] Conners CK. Manual for the Conners1 Continuous Performance Test-II Tonawanda. Now York, NY: Multi-Heath Systems (2002).

[67] Duchesne M Mattos P. Normalization of a computerized visual attention test (TAVIS). Arq Neuropsiquiatr. (1997) 55:62–9. 9332562 10.1590/s0004-282x1997000100010

[68] Agessi LM Villa TR Carvalho D de S Pereira LD. Auditory processing in children with migraine: a controlled study. Neuropediatrics. (2017) 48:123–6. 10.1055/s-0037-1598046 28122382

[69] Salem H Vivas D Cao F Kazimi IF Teixeira AL Zeni CP. ADHD is associated with migraine: a systematic review and meta-analysis. Eur Child Adolesc Psychiatry. (2018) 27:267–77. 10.1007/s00787-017-1045-4 28905127

[70] Langdon R DiSabella M Strelzik J Fletcher A. Pediatric migraine and academics. Curr Pain Headache Rep. (2020) 24:40. 10.1007/s11916-020-00869-5 32529391

[71] Mazzone L Vitiello B Incorpora G Mazzone D. Behavioural and temperamental characteristics of children and adolescents suffering from primary headache. Cephalalgia Int J Headache. (2006) 26:194–201. 10.1111/j.1468-2982.2005.01015.x 16426275

[72] First MB. Diagnostic and Statistical Manual of Mental Disorders: DSM-IV. Washington, DC: American Psychiatric Publishing (1994).

[73] Woolger C. Wechsler Intelligence Scale for Children-(WISC-III). In: Understanding Psychological Assessment. Boston, MA: Springer (2001). p. 219–33.

[74] Beery KE. Beery VMI: The Beery-Buktenica Developmental Test of Visual-Motor Integration. Minneapolis, MN: Pearson (2004).

[75] Henderson SE. Movement assessment battery for children. Psychol Corporat. (1992).

[76] Parisi P Verrotti A Paolino MC Ferretti A Raucci U Moavero R. Headache and attention deficit and hyperactivity disorder in children: common condition with complex relation and disabling consequences. Epilepsy Behav EB. (2014) 32:72–5. 10.1016/j.yebeh.2013.12.028 24495865

[77] Scherder EJA Rommelse NNJ Bröring T Faraone SV Sergeant JA. Somatosensory functioning and experienced pain in ADHD-families: a pilot study. Eur J Paediatr Neurol EJPN Off J Eur Paediatr Neurol Soc. (2008) 12:461–9. 10.1016/j.ejpn.2007.11.004 18262449

[78] Treister R Eisenberg E Demeter N Pud D. Alterations in pain response are partially reversed by methylphenidate (Ritalin) in adults with attention deficit hyperactivity disorder (ADHD). Pain Pract Off J World Inst Pain. (2015) 15:4–11. 10.1111/papr.12129 24134430

[79] Goadsby PJ Lipton RB Ferrari MD. Migraine–current understanding and treatment. N Engl J Med. (2002) 346:257–70. 10.1056/NEJMra010917 11807151

[80] Akerman S Goadsby PJ. Dopamine and migraine: biology and clinical implications. Cephalalgia Int J Headache. (2007) 27:1308–14. 10.1111/j.1468-2982.2007.01478.x 17970991

[81] Matsutani K Tsuruoka M Shinya A Furuya R Kawawa T. Stimulation of the locus coeruleus suppresses trigeminal sensorimotor function in the rat. Brain Res Bull. (2000) 53:827–32. 10.1016/s0361-9230(00)00426-3 11179850

[82] Araujo GC Dodd JN Mar S. Cognitive and emotional functioning in pediatric migraine relative to healthy control subjects. Pediatr Neurol. (2020) 111:35–6. 10.1016/j.pediatrneurol.2020.06.003 32951654

[83] Powers SW Coffey CS Chamberlin LA Ecklund DJ Klingner EA Yankey JW. Trial of amitriptyline, topiramate, and placebo for pediatric migraine. N Engl J Med. (2017) 376:115–24. 10.1056/NEJMoa1610384 27788026 PMC5226887

[84] Villa TR Agessi LM Moutran ARC Gabbai AA Carvalho D de S. Visual attention in children with migraine: the importance of prophylaxis. J Child Neurol. (2016) 31:569–72. 10.1177/088307381560149 26323497

